# Medical Justice and Liberality—New Pathological Phenomenon

**Published:** 1829-04-01

**Authors:** 


### XVII.

### MEDICAL JUSTICE AND LIBERALITY—NEW PATHOLOGICAL PHENOMENON.

The spirit of the FREE PRESS has now given ample proofs of its existence in every direction—and liberalism, as it is miscalled, shews itself in its true colours—freedom from every sentiment of truth, candour, or justice! These remarks will be amply illustrated by a late Coroner's inquest on two children who died of scarlatina, in the parish of St. James's, Westminster. They had been attended by Mr. Davis, of King Street, St. James's, and Mr. Duchez, of Air Street, Piccadilly. The latter gentleman served his apprenticeship with Mr. Alcock, and had, in the parish work-house, a long and extensive opportunity of seeing and treating both measles and scarlatina. On these complaints he read a very able paper in the Westminster Medical Society, during the present session. Dr. Merriman was ultimately called in, in the cases under consideration, and approved of the treatment pursued by Mr. Duchez and Mr. Davis. But, from what cause we shall not pretend to say, the parents of the children were excited against the two first medical attendants, and Mr. Healey, Mr. Tuson, and Mr. Dermott, were requested to examine the bodies of the children anatomically, *in order to ascertain whether proper medicines had been administered!* "The opinions of these gentlemen were, that, had proper remedies been applied, the lives of these children might have been saved." [*Times Newspaper, 27th Dec.*] Dr. Merriman, who saw the children during life—who had communication with Mr. Duchez, and consequently who was made acquainted with the treatment—did not object to the means employed—and did not consider that the children died of improper treatment.

Now we call upon the anatomical investigators, in this case, to inform their brethren by what means, and upon what pathological principle, they were able to ascertain that, had proper remedies been employed, these children would not have died of scarlatina. What will foreign nations say to the medical profession of this country, when they see such monstrous instances of absurdity and cruelty proceed from surgeons when employed in the solemn act of juridical investigation! If the report in the TIMES be correct, then we say that it is absolutely impossible for the anatomical witnesses to support or to excuse the illiberality—not to say *injustice* of their report. Was there ever such an idea broached before, as that the scalpel will detect the non-application of proper medicines!!! If indeed Mr. Duchez had administered a dose of arsenic and poisoned the little patients, at once, then anatomy and chemistry might have proved that *improper* medicines had been given by which the children came by their death. But for anatomy to be appealed to, for the purpose of proving that proper medicines had *not* been administered, and that had they been exhibited, life would have been saved, exceeds all credibility, and can only be explained by the dreadful state of public and private feeling universally diffused among the members of a once liberal and respectable profession!

Surely these transactions will open the eyes of the three corporate bodies, and induce them to unite in an application to parliament for one strong and uniform system of government in medical society. Were there such a head, with legal powers to enforce proper regulations, and punish by expulsion, those who broke the laws of decorum and medical ethics, the profession would soon assume a new and a better aspect. Till such a measure is adopted—till such powers are sought, obtained, and exercised, the spirit of illiberality and censoriousness will, we fear, continue—and the medical profession, in this country, will be an object of wonder over every civilized nation on earth.

P. S. We have been informed by a friend, after the above was written, that Mr. Dermott publickly declared that he had signed the anatomical report *without reading it;* and that he disapproved of the said report, when his attention was called to the illiberal and unjust principles involved in it. If this be the case, Mr. Dermott should shew who was the framer of the report, in order that professional indignation may be directed towards him. This inquest may teach Mr. Dermott a lesson, not to affix his signature again to any document tending to calumniate a brother-practitioner, without being very sure of the grounds on which the document is framed. This negligence, in itself, is highly censurable.

---

